# Supplementary material for: KRAS Mutations Impact Clinical Outcome in Metastatic Non-Small Cell Lung Cancer
Source: Cancers (Basel). 2022 Apr 20;14(9):2063. doi: 10.3390/cancers14092063 (PMC9103674; doi:10.3390/cancers14092063)
Supplement: Supplementary file 1 [file cancers-14-02063-s001.zip › cancers-1663225-supplementary.pdf]

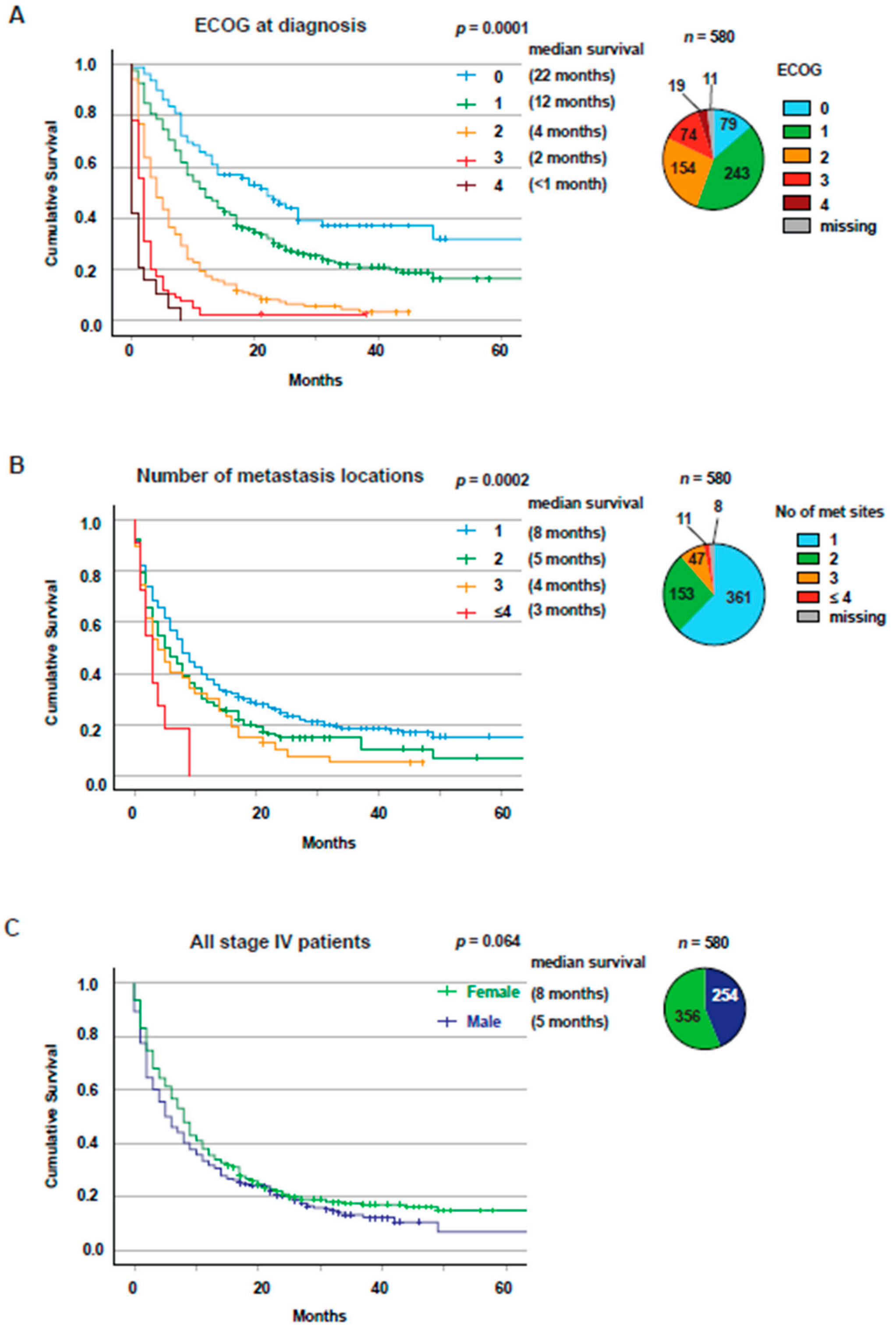

Figure S1. (**A**). Kaplan-Meier estimates comparing overall survival for the full cohort stratified on ECOG PS. Pie chart showing patient distribution between ECOG stages. (**B**). Kaplan-Meier estimates comparing overall survival for the full cohort stratified on number of metastasis locations. Pie chart showing patient distribution between number of metastasis locations. (**C**). Kaplan-Meier estimates comparing overall survival for the full cohort stratified on gender. Pie chart showing patient distribution between male and female. ECOG: Eastern Cooperative Oncology Group; PS: Performance Status.

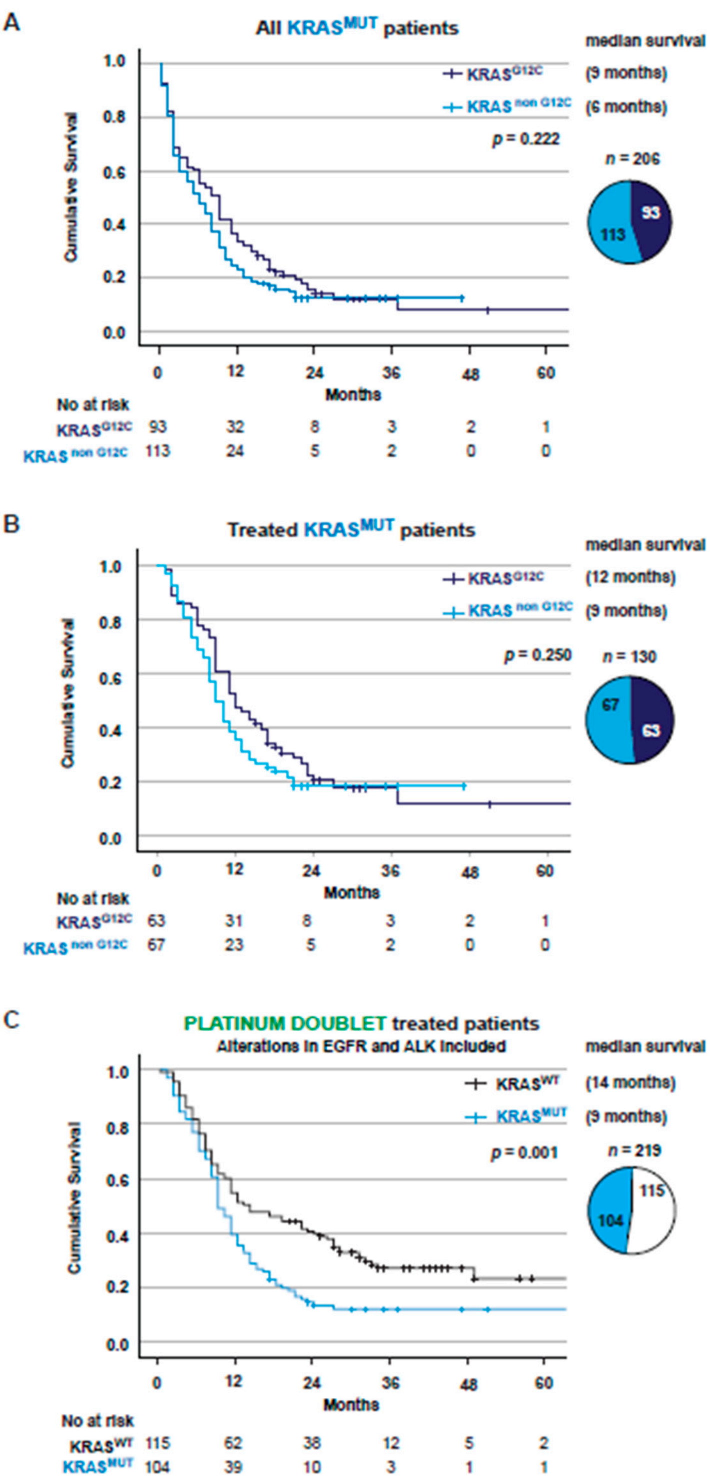

Figure S2. (**A**). Kaplan–Meier estimates comparing overall survival for all KRASMUT stratified on KRASG12C vs. KRASnon G12C. Pie chart showing patient distribution between KRASG12C and KRASnonG12C. (**B**). Kaplan–Meier estimates comparing overall survival for all KRASMUT patients receiving treatment with platinum doublet stratified on KRASG12C vs. KRASnon G12C. Pie chart showing patient distribution between KRASG12C and KRASnon G12C. (**C**). Kaplan–Meier estimates comparing overall survival for all receiving treatment with platinum doublet stratified on KRASWT vs. KRASMUT. Pie chart showing patient distribution between KRASWT and KRASMUT

Table S1. (Related to Figure S2C), Characteristics of all PT treated patients.

Characteristics all Platinum Doublet treated	WT	KRAS MUT
	n (%)	*n* (%)
Total *n* = 219	115 (52,5)	104 (47,5)
Age in years, median (range)	69 (24–84)	70 (35–83)
Sex		
Male	49 (42,6)	35 (33,7)
Female	66 (57,4)	69 (66,3)
Smoking history		
Current smoker	38 (33,0)	37 (35,6)
Former smoker	53 (46,1)	60 (57,7)
Never smoker	23 (20,0)	7 (6,7)
Missing	1 (0,9)	0
Perfomance status		
ECOG 0	22 (19,1)	24 (23,1)
ECOG 1	66 (57,4)	60 (57,7)
ECOG 2	25 (21,7)	20 (19,2)
ECOG 3	2 (1,7)	0
ECOG 4	0	0
Missing	0	0
Histology		
Adenocarcinoma	94 (81,8)	88 (8,6)
NSCLC NOS	9 (7,8)	15 (14,4)
Squamous cell carcinoma	12 (10,4)	1 (1,0)
Mutation status		
None known	77 (67,0)	
ALK	6 (5,2)	
EGFR	18 (15,7)	
BRAF	6 (5,2)	
Other	6 (5,2)	
1RET	2 (1,7)	
KRAS submutation		
G12A		6 (5,8)
G12C		46 (44,2)
G12D		10 (9,6)
G12V		20 (19,2)
Others		22 (19,2)
At last follow up		
Alive	32 (27,8)	14 (13,5)
Deceased	83 (72,2)	90 (86,5)
Survival		
Median survival (months)	14	9
No. of metastatic locations at diagnosis		
1	79 (68,7)	72 (69,2)
2	26 (22,6)	22 (57,7)
3	6 (5,2)	7 (6,7)
>3	1 (0,9)	3 (2,9)
Missing	3 (2,6)	0

Table S2. (Related to Figure 5A). Characteristics of all patients treated with first-line ICB.

Characteristics Immune checkpoint blockade monotherapy	WT	KRAS
	n (%)	*n* (%)
Total *n* = 37	*n* = 17(45,9)	*n* = 20 (54,1)
Age in years, median (range)	70 (57–83)	69 (46–78)
Sex		
Male	6 (35,3)	12 (60)
Female	11 (64,7)	8 (40)
Smoking history		
Current smoker	7 (41.2)	7 (35)
Former smoker	9 (52,9)	12 (60)
Never smoker	1 (5,9)	1 (5)
Missing	0	0
Perfomance status		
ECOG 0	3 (17,6)	4 (20)
ECOG 1	8 (47,1)	13 (65)
ECOG 2	6 (35,3)	3 (15)
ECOG 3	0	0
ECOG 4	0	0
Missing	0	0
Histology		
Adenocarcinoma	14 (82,4)	19 (95)
NSCLC NOS	2 (11,8)	1 (5)
Squamous cell carcinoma	1 (5,9)	0
Mutation status		
None known	14 (82,4)	
ALK	0	
EGFR	1 (5,9)	
BRAF	1 (5,9)	
NRAS	1 (5,9)	
ROS1	0	
1RET	0	
KRAS submutation		
G12A		2 (10)
G12C		8 (40)
G12D		1 (5)
G12V		2 (10)
G13D		2 (10)
Q61H		2 (10)
Other		3 (15)
PD-L1 status		
High TPS ≥50%	13 (76,5)	20 (100)
Low TPS 1–49%	3 (17,6)	0
Missing	1 (5,9)	0
At last follow up		
Alive	2 (11,8)	10 (50)
Deceased	15 (88,2)	10 (50)
Survival		
Median survival (months)	6	23
No. of metastatic locations at diagnosis		
1	14 (82,4)	10 (50)
2	2 (11,8)	9 (45)
3	1 (5,9)	1 (5)

Table S3. Multivariate analyses.

	**ALL STAGE IV**		
	**HR**	**95%CI**	***p***
KRAS mutation	1.472	1.207–1.769	<0.001
ECOG >1	3.175	2.622–3.845	<0.001
Gender (female)	0.840	0.700–1.008	0.061
Smoking	1.021	0.803–1.297	0.867
	All PT with EGFR and ALK excluded		
	HR	95%CI	*p*
KRAS mutation	1.564	1.124–2.177	0.008
ECOG >1	1.906	1.307–7.779	<0.001
Gender (female)	0.957	0.690–1.328	0.794
Smoking	1.089	0.608–1.952	0.774
	All ICB treated		
	HR	95%CI	*p*
KRAS mutation	0.349	0.148–0.822	0.016
ECOG >1	0.389	0.165–0.963	0.041
Gender (male)	1.459	0.637–3-343	0.372
Smoking	1.263	0.165–9.658	0.882
	KRAS MUT PD-L1 status		
	HR	95%CI	*p*
PD-L1	0.993	0.986–0.999	0.028
ECOG >1	2.470	1.470–4.273	0.001
Gender (male)	1.026	0.632–1.166	0.918
Smoking	1.212	0.421–3.491	0.721
	KRAS WT PD-L1 status		
	HR	95%CI	*p*
PD-L1	1.001	0.995–1.007	0.780
ECOG >1	2.865	1.938–4.236	<0.001
Gender (female)	0.926	0.642–1.343	0.918
Smoking	0.743	0.473–1.168	0.198
